# Organising Pneumonia in a Patient Following Obinutuzumab for Follicular Lymphoma

**DOI:** 10.1002/rcr2.70258

**Published:** 2025-06-26

**Authors:** Viral Nanda, Huw Ellis, Harshana Bandara

**Affiliations:** ^1^ Northwest Lung Centre Wythenshawe Hospital, Manchester University NHS Foundation Trust Manchester UK

**Keywords:** clinical respiratory medicine, inflammation, interstitial lung disease, lung fibrosis, pharmacogenetics

## Abstract

Hereby presenting a patient with follicular lymphoma, treated with obinutuzumab subsequently developing organising pneumonia. The case highlights newer generation culprits causing pulmonary complications and the importance of vigilant imaging survey and timely treatment to prevent the fibrotic progression in these patients.

## Graphical abstract

We present a patient with follicular lymphoma, treated with obinutuzumab who subsequently developing organising pneumonia. The case highlights newer generation of culprits causing pulmonary complications and the importance of vigilant imaging survey and timely treatment to prevent fibrotic progression in these patients.

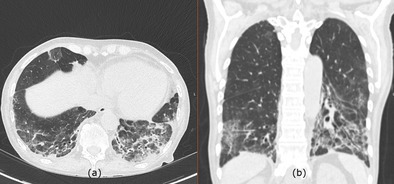

A 70‐year‐old female with a history of follicular lymphoma previously treated with chemotherapy including rituximab, bendamustine was subsequently started on obinutuzumab (a humanised anti‐CD20 monoclonal antibody) due to progression of the disease was treated with 12 cycles of obinutuzumab. Since then, she started complaining of worsening shortness of breath and cough 3 months after stopping the treatment along with weight loss. Repeat computed tomography (CT) of the chest, abdomen and pelvis was performed and revealed remission of lymphoma. However, the parenchymal changes of the lung were suggesting organising pneumonia (OP) (Figure 1). Evaluation of the routine and the atypical infections did not reveal any infective cause during that time and the procalcitonin was also negative. The patient was scheduled for bronchoscopy but unfortunately could not attend as she was unwell with a chest infection during the scheduled date. There were no features to suggest connective tissue diseases and the autoimmune profile was negative. In view of worsening symptoms, she was started on prednisolone 25 mg based on her weight in view of the CT changes. A multi‐disciplinary team meeting along with a thoracic radiologist concluded the diagnosis of OP based on the radiological features. Following the steroids, she developed infections with pseudomonas and was treated with anti‐pseudomonal treatment while continuing the low‐dose steroids. The patient responded well to the steroids and improved significantly in terms of her breathlessness and cough, and a repeat scan while the patient was on 10 mg prednisolone showed significant improvement in terms of her OP. The CT scan following the steroid treatment showed almost complete resolution of the organising pneumonia with minimal residual reticulation (Figure 2).

**FIGURE 1:**
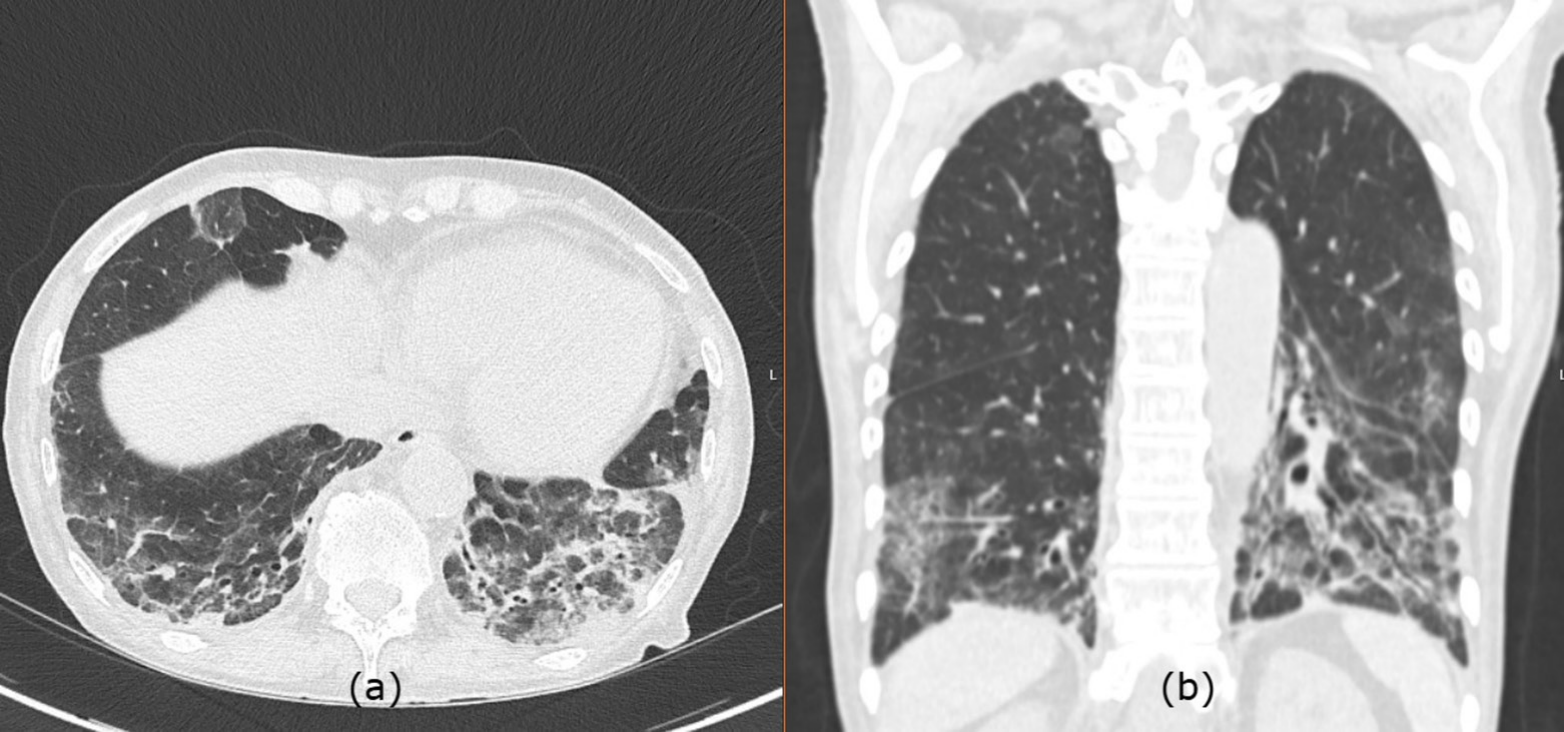
Initial CT scan after treating with obinutuzumab showing lower zone predominant peri‐broncho‐vascular consolidation with admixed ground‐glass and volume loss with tractional bronchiectasis which are evident in both axial (a) and coronal (b) images, suggesting organising pneumonia.

**FIGURE 2:**
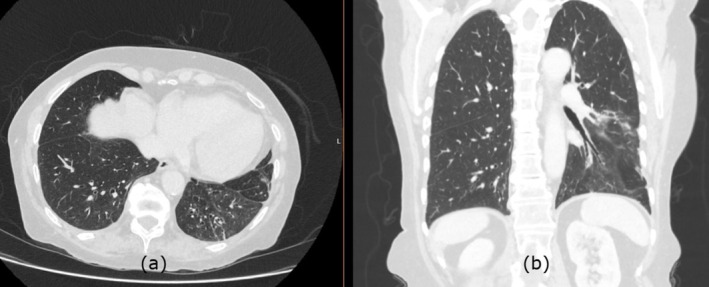
The CT scan following the treatment with steroids showing near complete resolution of the left lower lobe consolidation with minimal residual reticulation both in axial (a) and coronal images (b).

There have been very few case reports highlighting the fact that obinutuzumab causes OP. Obinutuzumab is an anti‐CD‐20 monoclonal antibody used for the treatment of follicular lymphoma along with bendamustine and rituximab [1]. It is also approved for the treatment of chronic lymphocytic leukaemia along with chlorambucil [2, 3]. The pathogenesis of drug‐induced OP remains incompletely understood but is due to an aberrant immune response leading to lung injury. In the context of obinutuzumab, its immunomodulatory effects might predispose patients to such complications [3]. Adverse events of the drug reported include infusion‐related reactions, infection, neutropenia, anaemia, thrombocytopenia and cardiac events [1]. There is very little literature so far about the drug causing OP and hence reporting this case becomes very important to consider in the future when patients are on this drug. Based on the temporal relationship, the classic radiology pattern, the absence of other causes of OP, and the response to the steroids with near normal resolution, we concluded that obinutuzumab has likely led to OP in this patient. However, we need to highlight the absence of biopsy and bronchoalveolar lavage as limitations.

## Author Contributions

Viral Nanda drafted the manuscript. Harshana Bandara revised the manuscript. All authors approved the final manuscript.

## Consent

The authors declare that written informed consent was obtained for the publication of this manuscript and accompanying images and attest that the form used to obtain the consent from the patient complies with the journal requirements as outlined in the author guidelines.

## Conflicts of Interest

The authors declare no conflicts of interest.

## Data Availability

Data sharing is not applicable to this article as no new data were created or analyzed in this study.
